# Predictors of Cognitive Function in Community-Dwelling Older Adults by Age Group: Based on the 2017 National Survey of Older Korean Adults

**DOI:** 10.3390/ijerph18189600

**Published:** 2021-09-12

**Authors:** Jinheum Kim, Eunjeong Cha

**Affiliations:** 1 Department of Applied Statistics, The University of Suwon, Hwaseong-si 18323, Korea; jinhkim@suwon.ac.kr; 2 Department of Nursing Science, The University of Suwon, Hwaseong-si 18323, Korea

**Keywords:** older adults, cognitive function, age group, electronic device-based activities, IADL

## Abstract

Owing to a growing older adult population, dementia is emerging as an important health issue. Given that maintaining cognitive functions is crucial for the prevention of dementia, this study aimed to identify the predictors of cognitive function in community-dwelling older adults, through a secondary data analysis of the 2017 National Survey of Older Koreans. A total of 9836 participants were classified into three age groups—young-old (65–74 years), old-old (75–84 years), and oldest-old (≥85 years)—and were separately analyzed using multiple linear regression models. The final model explained 28.0%, 35.0%, and 37.0% of variance in cognitive function in the three age groups, respectively. The most potent predictors of cognitive function in the young-old were electronic device-based activities, instrumental activities of daily living (IADL), and nutrition management; the predictors for the old-old group were electronic device-based activities, IADL, and dementia screening, and those for the oldest-old group were frequency of contact with acquaintances, traveling, and religion. Thus, age group-specific interventions are needed to effectively promote cognitive function among older adults. Digital literacy education, use of community-based elderly welfare programs, opportunities for social interactions, and physical activities can help older adults in maintaining a functional status and muscle strengthening.

## 1. Introduction

An analysis of the Organization for Economic Co-operation and Development (OECD) statistics over the last 50 years from 1970 to 2018 revealed that South Korea has the fastest low fertility and aging rate among 37 OECD countries [1]. South Korea officially became an “aged society” in 2018, 18 years after being designated as an “aging society” in 2000, and the older adult population (≥65 years) accounted for 15.7% of the total population in 2020 [2]. At this pace, the OECD predicts that South Korea will become a “super-aged society,” defined as a society with older adult population of 20% or higher, by 2026, eight years after becoming an aged society [1]. With a growing older adult population, active ongoing research is being conducted on various aspects of health, including geriatric degenerative diseases and chronic diseases. Of these health problems, dementia is one of the most rapidly growing health problems. The World Health Organization (WHO) reports that approximately 50 million people worldwide suffer from dementia, with the number projected to increase to 152 million by 2050 due to increasing aging population [3]. In South Korea, 750,000 people aged 65 years or older had dementia as of 2018, and the prevalence of dementia is forecasted to surpass one million in 2024 [4].

Dementia does not occur suddenly in individuals with normal cognitive function but develops with gradual cognitive decline [5], with patients demonstrating characteristic symptoms of gradual cognitive decline in various areas, including memory, learning, orientation, language, comprehension, and judgment [6]. Approximately 10–15% of patients with mild cognitive impairment (MCI), a condition considered an antecedent of dementia, are diagnosed with dementia every year [7], and delaying the onset of dementia by two years is reported to reduce the severity of dementia and the prevalence of dementia to 80% of the current prevalence [8]. Thus, focusing on older adults’ cognitive functions is important in that maintaining good cognitive functions in community-dwelling older adults enables the prevention of dementia.

Accordingly, changes in cognitive function in older adults and their associated factors have attracted much attention in recent years [9,10]. Previous studies suggest that the time of onset of cognitive decline, degree of decline, and rate of decline among older adults vary widely [9,11] and differ across individuals [12]. Furthermore, previous studies have reported that older adults’ cognitive functions are influenced by age, sex, and education level [13,14,15], as well as the presence of a spouse, activities of daily living (ADL), nutrition, physical activity, drinking, exercise, body weight, social relationships, and depression [14,15,16,17,18]. However, these results have been inconsistent across studies, and cognitive prediction models for older adults proposed in past studies include both patients with cognitive impairment and patients with dementia, which hinder the generalization of the models for the prevention of cognitive decline in community-dwelling older adults [19]. Therefore, studies on community-dwelling older adults are required.

As a result of the broadening age span of the older adult population due to the increased lifespan, there is a mounting interest in the diversity of age groups within the older adult population. If age 65 is considered the beginning of older adulthood, the age gap among older adults would be between 25 and 30 years [20]. According to the 2017 National Survey of Older Koreans (NSOK), the composition and features of the older adult population (≥65 years) have changed over the past 10 years. The percentage of older adults aged 80 years or older continuously rose from 16.0% in 2008 to 21.7% in 2017, and older adults’ general and environmental characteristics, such as education level, living arrangement, and household structure, also changed [21]. Thus, viewing the older adult population as a single group may decrease the accuracy of sociodemographic factors, health status, health behaviors, and environmental factors that affect older adults’ cognitive functions.

Accordingly, this study subdivided the older adult population into: young-old (65–74 years), old-old (75–84 years), and oldest-old (≥85 years) in accordance with the classification by Crews and Zavotka [22] and attempted to identify age group-specific modifiable factors that can help prevent cognitive decline in older adults using the raw data of the 2017 NSOK, a survey conducted by the Ministry of Health and Welfare (MOHW) and Korea Institute for Health and Social Affairs (KIHASA) on a nationally representative community-dwelling older adult sample in Korea [23]. Based on these findings, we aimed to present foundational data for developing systematic and effective age group-specific intervention strategies for maintaining the cognitive functions of community-dwelling older adults.

The specific objectives were as follows: (1) examine the differences in cognitive functions according to sociodemographic characteristics, health status, health behaviors, functional state, social relations, and activities by age group. (2) Identify predictors of cognitive function after accounting for sociodemographic characteristics by age group

## 2. Materials and Methods

### 2.1. Study Design and Data Source

This is a secondary data analysis study of the 2017 NSOK conducted by the MOHW and KIHASA [24] to identify the predictors of cognitive functions by age group among community-dwelling older adults. The NSOK is a government-approved statistical data collected every three years in accordance with Article 5 (Implementation of the NSOK) of the Welfare of the Senior Citizens Act (January 2007), which is representative of the current standard of living and needs of older adults aged 65 years or older in communities nationwide. The objective of the NSOK is to predict changes in Korean older adults to contribute to developing policies that improve older adults’ quality of life and to devise measures to deal with an aged society [24].

### 2.2. Participants

Raw data from the 2017 NSOK conducted on older adults aged 65 years or older nationwide by KIHASA was used. The sample for the raw data was extracted via stratified two-stage cluster sampling, where the study population was clustered into 17 cities and provinces in the first stage, and 9 provinces and Sejong Special Autonomous City (excluding 7 special and metropolitan cities) were partitioned into ‘dong’ region and ‘eup or myeon’ region in the second stage, followed by sampling of older adults from each of the 17 cities and provinces proportionate to the square root of the number of older adults registered in the 2015 Census [24]. Thus, each sample had both household and individual weights determined by a complex sample design. Statistical analyses were conducted by considering these features.

### 2.3. Measurements

In this study, the study variables based on the survey items of the 2017 NSOK data by the KIHASA were defined as follows:

#### 2.3.1. Sociodemographic Characteristics

Sex, education level, spouse, employment status, annual household income, and household structure were used as sociodemographic characteristics in this study. The economic status of older adults was divided into quintiles based on their annual household income [21]. The criteria for the quintiles were: the 1st quintile: (≤10.08 million KRW), the 2nd quintile: (10.09–15.08 million KRW), the 3rd quintile: (15.09–23.31 million KRW), the 4th quintile: (23.32–37.89 million KRW), and the 5th quintile: (>37.89 million KRW). Household structure for older adults was divided into “older adult with spouse,” “older adult with children,” and “single older adult” [21].

#### 2.3.2. Health Status

Health status included perceived health, number of chronic diseases, and depression. Perceived health was determined using the older adults’ response on one item about their self-rated health, rated on a five-point scale: (1) very healthy, (2) healthy, (3) so-so, (4) not healthy, and (5) very unhealthy [21]. In this study, the responses were categorized into “healthy” (1 and 2), “so-so” (3), and “not healthy” (4 and 5). The number of chronic diseases was determined based on an item asking about objective health status with the names of 30 diseases, and the participants were asked to indicate the total number of chronic diseases they suffered from, since last three months or longer and were diagnosed by a physician [21]. The responses were divided into “0”, “1”, or “≥2”. Depression was measured using the Korean version of the Geriatric Depression Scale Short Form (GDS-SF). The GDS-SF contains 15 items with a possible total score of 15 [25]. A higher score indicates a higher level of depression; a score of 7 or lower indicates “normal,” and 8 or higher indicates “depressed” [26]. Cronbach’s α was 0.88 in a prior study [26] and 0.89 in this study.

#### 2.3.3. Health Behaviors

Health behaviors included nutrition management, smoking, drinking, exercise, and dementia screening. Nutrition management was measured using the 10-item “Determine Your Nutrition Health” developed by the Nutrition Screening Initiative (NSI). The items were differentially weighted for a total possible score of 21. A score of 0–2 indicated “low risk,” score of 3–5 indicated “moderate risk,” and a score of 6 or higher indicated “high risk” [27]. Drinking (over the past year) was divided into “I don’t drink,” “moderate drinking” (7 or fewer drinks per week), and “heavy drinking” (more than 7 drinks per week) [21]. Smoking (current) and dementia screening were used as binary responses (yes or no) [21]. Exercise was divided into “I don’t exercise,” “below recommendation’’ (<150 min/week), and “meet recommendation” (≥150 min/week) [21].

#### 2.3.4. Functional Status

Functional status was determined based on instrumental activities of daily living (IADL) and muscle training. The IADL were measured using 10 items, with a higher score indicating lower performance [28]. Cronbach’s α was 0.94 in the previous study [23] and 0.89 in this study. A total IADL score of below 10 indicates “need assistance,” and a score of 10 indicates “completely independent.” Muscle training was determined based on whether the participant could perform five repetitions of sit to stand (standing up from a seated position on a chair or bed) [21].

#### 2.3.5. Social Relations

Social relations were determined based on the frequency of contact with family, relatives, and friends in the past year. The responses were divided into “rarely,” “fewer than once a week,” and “once or more per week” [21].

#### 2.3.6. Activities

Activities included watching TV, traveling, leisure, learning, club, and social activities, religion, and use of senior centers and electronic devices [21]. Traveling, leisure, learning, and club activities, religion, and use of senior citizen centers were assessed as binary responses (yes or no). Watching TV was divided into “<5 h/day” and “≥5 h/day,” and social activities was divided into “none,” “<1/week” and “≥1/week”. Use of electronic devices was defined as the number of activities performed using an electronic device and was divided into “0”, “1”, or “≥2”.

#### 2.3.7. Cognitive Function

Cognitive function was measured using the Korean version of the Mini-Mental State Examination for Dementia Screening (MMSE-DS). The MMSE-DS comprises 19 items measuring general cognitive function, including orientation, memory, and attention, with a total possible score of 30 [29]. In this study, we used the MMSE score to represent the participants’ cognitive function. The Cronbach’s α of this tool was 0.82 in a prior study [29] and 0.80 in this study.

### 2.4. Ethical Considerations

The 2017 NSOK was approved by the National Statistical Office (Approval No. 11771). In accordance with the “Bioethics and Safety Act,” the NSOK was approved by the Institutional Review Board of the KIHASA (IRB No.17-034-00) for the protection of research participants. This study was approved for review by the Institutional Review Board at the University to which researchers belong (USW IRB/2011-045-02). To use the 2017 NSOK data for research, we joined the Health and Welfare Data Portal, disclosed the researcher’s information and purpose of use, and agreed to comply with the management rules to download the 2017 NSOK raw data, questionnaire, and code book. Data are available with permission from the Health and Welfare Data Portal https://data.kihasa.re.kr/ (1 August 2021).

### 2.5. Data Analysis

The raw data used in this study were collected using a complex sampling design and were weighted individually and house-wise. The data were analyzed using SAS 9.4 software (SAS Institute Inc., SAS/STAT 9.4 User’s Guide, Cary, NC, USA) depending on the purpose of analysis. The participants were divided into three groups according to their age, following the classification criteria proposed by Crews and Zavotka [22], in order to identify age-specific factors affecting cognitive function.

(1) Participants’ sociodemographic characteristics were presented as real numbers and percentages for the total population and each age group using the SAS SURVEYFREQ procedure. The differences in sociodemographic characteristics across age groups were analyzed using the Rao-Scott $\chi^{2}$-test.
(2) Participants’ cognitive function (MMSE) were presented as mean and standard deviation by age group and category of each variable using the SAS SURVEYMEANS procedure. Differences in cognitive function among the levels of each categorical variable were analyzed with t-test or F-test using the SAS SURVEYREG procedure.
(3) The predictors of cognitive function by age group were identified with multiple linear regression analysis using the SAS SURVEYREG procedure.

## 3. Results

### 3.1. Participants’ Sociodemographic Characteristics

Of the 9836 participants, 3936 were male and 5900 were female. Regarding education level, 23.5% were uneducated, 34.1% had elementary graduation, 34.7% had middle or high school graduation, and 7.7% had college graduation or higher. The majority (63.6%) of the participants had spouses, and 30.4% were employed. Regarding annual household income, 20.2% were in the 1st quintile (≤10.08 million KRW), while 19.8% were in the 5th quintile (>37.89 million KRW). The most common household structure for older adults was living with a spouse (48.6%), followed by living with children (27.4%) and living alone (24.0%). There were significant differences in all sociodemographic characteristics among the age groups (Table 1).

### 3.2. Participants’ Level of Cognitive Function and the Differences among Categories of Each Variable by Age Group

Table 2 shows the differences in cognitive function according to sociodemographic characteristics, health status, health behavior, functional status, social relations, and activities by age group.

The mean (± standard deviation) of MMSE was 26.25 (±3.09) in the young-old group, 24.27 (±4.02) in the old-old group, and 21.79 (±4.90) in the oldest-old group, and the differences among the age groups were statistically significant (F = 444.64, *p* < 0.001).

All three groups significantly differed in cognitive function according to sex, education level, presence of spouse, and household structure. Cognitive function was higher among older men than among older women, among those with a spouse, and among those who only lived with a spouse. However, the differences in cognitive function according to employment status and annual household income were not significant in the oldest-old group.

Regarding health status, all three groups significantly differed in their cognitive function according to perceived health and depression. The difference in cognitive function according to the number of chronic diseases was significant in the young-old and old-old groups.

Regarding health behaviors, cognitive function was higher among those at low risk in terms of nutrition management, those who drank (≥1 drink per week), and those who engaged in more exercise in all three groups. The difference in cognitive function according to dementia screening was significant in the old-old and oldest-old groups, and the difference in cognitive function according to smoking was significant in the young-old and old-old groups.

Regarding functional status, cognitive function was significantly higher with independent IADL and ability to perform muscle training in all three groups.

Regarding social relations, cognitive function was significantly higher with a greater frequency of contact with relatives and friends in all three groups.

Regarding activities, cognitive function was significantly higher with more frequent traveling, leisure activities, learning activities, religious activities, club activities, social activities, and electronic device-based activities in all three groups. In the young-old and old-old groups, cognitive function was high among those who did not use a senior center and watched TV for less than five hours a day.

### 3.3. Predictors of Cognitive Function by Age Group

We performed multiple linear regression after accounting for sociodemographic characteristics using the SAS SURVEYREG procedure, and the final model was selected based on the backward elimination strategy. The variables were extracted with a significance level (α) of 0.01.

The variation inflation factor (VIF) was computed to test for multicollinearity. The VIF was below the cutoff of 10 in the young-old group (1.00–1.43), the old-old group (1.02–1.66), and the oldest-old group (1.05–1.48), confirming the absence of multicollinearity that occurs when there is a high correlation among the predictors.

The backward elimination method was used to remove the most statistically insignificant variables from the initial model (including all the predictors) one at a time. The removal process was completed whenever the largest *p*-value obtained was less than 0.01, the variable selection criterion, and this model was considered to be the final model. The sociodemographic variables, however, were defined as control variables and were included in the final model regardless of their statistical significance. The predictors of cognitive function for each age group are shown in Table 3.

The final model explained 28.0% (F = 58.47, *p* < 0.001), 35.0% (F = 83.78, *p* < 0.001), and 37.0% (F = 25.08, *p* < 0.001) of the variance in cognitive function in the young-old, old-old, and oldest-old groups, respectively.

First, the predictors of cognitive function in the young-old group were nutrition management, dementia screening, IADL, ability to perform muscular exercise, frequency of contact with friends, traveling, club activities, use of senior citizen centers, electronic device-based activities, gender, and education. Of these twelve predictors, electronic device-based activities, IADL, and nutrition management were found to be the most potent predictors, along with gender and education. Second, the predictors of cognitive function in the old-old group were dementia screening, IADL, ability to perform muscular exercise, frequency of contact with family and relatives, social activities, religion, electronic device-based activities, gender, education, presence of a spouse, employment status, and household status. Of these twelve predictors, electronic device-based activities, IADL, and dementia screening were the most potent predictors in the order described, besides gender, education, presence of a spouse, employment status, and household status. Third, the predictors of cognitive function in the oldest-old group were the ability to perform muscular exercise, frequency of contact with friends, traveling, religion, and education. Of these five predictors, frequency of contact with friends, travel, and education were the most potent predictors.

## 4. Discussion

In this study, we classified older adults into young-old, old-old, and oldest-old and aimed to identify the factors of health status, health behaviors, functional status, social relations, and activities that predict cognitive function in each age group after controlling for sociodemographic characteristics using the 2017 NSOK data conducted by the KIHASA.

There were statistically significant differences in sociodemographic characteristics among the three age groups. These were women, less-educated individuals, individuals without a spouse, older adults living alone with advancing age, and the number of employed older adults and annual household income decreased with increasing age. These trends are in line with those of previous studies [30,31]. Moreover, cognitive function significantly differed among the age groups, where it declined with advancing age, which is supported by many previous studies [9,32]. Therefore, as sociodemographic characteristics and cognitive functions vary significantly among age groups, age group-specific analyses are needed to identify the predictors of cognitive function among older adults.

Next, we discussed the major factors that predict cognitive function in each age group.

First, the most potent predictor of cognitive function in the young-old group was electronic device-based activities, which is in line with previous findings that older adults who use mobile devices demonstrate better cognitive functions [33] and that owning a computer or cell phone slows down the rate of cognitive decline [34]. A study that analyzed the predictors of cognitive function in older adults [35] also reported that electronic device-based activities were the strongest predictor of cognitive function in older adults. The IADL was the second-most potent predictor, which is consistent with previous findings [32,34]. Considering the finding that restriction of IADL increases the risk for cognitive decline and that individuals who are dependent on IADL are likely to begin to show cognitive decline three years later [36], proactively identifying older adults who need assistance with IADL based on the statements of their families or frequent contacts would help manage older adults at risk for cognitive decline. The third-most potent predictor was nutrition management, but it was only significant in the young-old group. This result is consistent with previous results that a healthy diet pattern lowers cognitive decline [18], and we also observed that the MMSE score was lower among those at high risk in terms of nutrition management in all three groups.

Second, the most potent predictors of cognitive function in the old-old group were electronic device-based activities, IADL, and dementia screening, and other predictors included muscle training, frequency of contact with family and relatives and friends, social activities, and religion. These findings are consistent with previous findings [13] that functional status and social relationships influence cognitive function in older adults. In the present study, dementia screening had a particularly strong influence in the old-old group. Although we cannot compare this result with the literature, given the lack of a relevant study, subsequent studies should examine whether early diagnosis through dementia screening and subsequent management has a positive impact, whether interest and awareness of the disease among those who seek dementia screening improve their cognitive functions, or whether dementia screening itself motivates older adults to maintain their cognitive function.

Third, frequency of contact with friends, traveling, religion, and ability to perform muscular movements were identified as the predictors of cognitive function in the oldest-old group. Although we cannot compare this with the literature, given the lack of studies conducted on the oldest-old age group (≥85 years), frequency of contact with friends was identified as an important predictor in this group. A systematic review on the relationship between social support and cognitive functions [37] reported that social support and cognitive function are positively associated, with emotional support serving as a key dimension. Therefore, these results suggest that active interactions with friends have a preventive effect against cognitive decline among oldest-old individuals. Traveling and religious activities were identified as positive predictors of cognitive function in the oldest-old group. Religion was also found to affect cognitive function in a previous study on community-dwelling older adults [38], so traveling and religious activities are expected to contribute to maintaining cognitive function in the oldest older adulthood, in which individuals engage in less social and physical activities. Moreover, considering the study findings that resistance exercises such as muscle training have positive effects on cognitive function [39], improving the ability to perform muscular exercise among the elderly would facilitate traveling and religious activities as well as enhance cognitive functions. Further studies shedding light on the predictors of cognitive function in the oldest-old population are needed in response to the rising oldest-old population (≥85 years), and implementation of age group-specific intervention programs would be effective in helping them maintain their cognitive functions and prevent dementia.

The results of this study highlight the need to develop cognitive intervention programs that reflect the predictors of cognitive function in each age group. Particularly, considering that electronic device-based activities were the most potent predictor of cognitive function in the young-old and old-old groups, digital literacy education should be provided for older adults early on to effectively maintain and enhance cognitive function, and educational approaches utilizing digital technology, such as smartphones, tablet computers, wearable devices, and remote monitoring technology, would be feasible. Furthermore, IADL was also a key predictor. Participation in leisure and social activities in older adulthood has been reported to prevent cognitive decline by increasing physical activity [40], so preventing IADL decline by boosting participation in community senior welfare center programs and senior citizen centers would be helpful. Additionally, community-based support measures for the oldest-old are needed to promote physical activities, leisure, hobbies, and social interactions in consideration of their general and environmental characteristics.

This study had several limitations. First, we could not examine various environmental factors that the participants experienced during their lifetime and the consequent changes in cognitive function due to the cross-sectional design. Second, this study was a secondary data analysis, so the study results should be interpreted with the features and limitations of the study instruments considered. Third, the proportion of uneducated older adults in our sample was relatively high (23.5%). As such, the distribution of the sample may be slightly different from that of the population; such a difference is a common weakness of secondary data analysis. Thus, if the proportion of uneducated older adults in our study is higher than the actual proportion, the estimates of education level presented in Table 3 may be somewhat underestimated. Nevertheless, regardless of age group, the effect of education level on cognitive function was found to be statistically very significant and to increase monotonically with education levels.

Despite these limitations, this study has several strengths. First, we included several independent variables from various areas after controlling for the sociodemographic characteristics to develop an optimal regression model and had adequate sample sizes for each age group, ensuring the representativeness of the study population. Second, we classified the older adults into specific age groups and shed light on the modifiable predictors, excluding sociodemographic factors, of cognitive function by age group to present useful data for developing effective interventions to maintain cognitive function in older adults.

## 5. Conclusions

The results showed that the predictors of cognitive function differed across age groups. Thus, maintaining cognitive function is important to prevent dementia in older adults, and age-group-specific approaches are needed to effectively intervene in older adults’ cognitive function. Hence, in the young-old group, the intervention should focus on electronic device-based activities, IADL, and nutrition management, while that in the old-old group should focus on electronic device-based activities, IADL, and dementia screening. In the oldest-old group, interventions for strength exercise, travel, religious activities, and frequent contact with acquaintances in light of general and environmental attributes are necessary.

The findings of this study can contribute to the development of intervention programs designed to prevent cognitive decline among older adults belonging to various age groups. Additionally, the final model in this study was determined using a variable selection method that helps to avoid overfitting and, therefore, increases the interpretability of the model owing to low complexity.

We suggest subsequent studies to utilize longitudinal designs to address the limitations of a cross-sectional study, through which they should identify the impact of the changes in general characteristics and environmental characteristics of older adults on their cognitive function. Moreover, as predictors of cognitive function changes in older adults in response to changes in culture and time, subsequent studies should address these issues. Additionally, intervention programs that reflect the predictors proposed in this study should be developed and implemented.

## Figures and Tables

**Table 1:** Sociodemographic characteristics by age group and homogeneity test between each variable and age group.

Variables	Categories	Total	Age Group			Rao-Scott	*p*-Value
			65–74	75–84	85+		
		*n* (% ^1^)	*n* (%)	*n* (%)	*n* (%)	*χ* ^2^	
Total		9836(100)	5194(58.2)	4011(34.1)	631(7.7)	-	-
Gender	Male	3936(42.4)	2139(59.3)	1585(34.1)	212(6.6)	10.15	0.0063
	Female	5900(57.6)	3055(57.3)	2426(34.2)	419(8.5)		
Education	Uneducated	2603(23.5)	835(35.7)	1440(48.3)	328(16.0)	605.24	<0.0001
	Elementary	3440(34.1)	1939(61.1)	1338(32.9)	163(5.9)		
	Middle-high	3145(34.7)	2057(70.5)	1002(26.1)	86(3.4)		
	College or above	648(7.7)	363(58.1)	231(31.9)	54(10.0)		
Spouse	No	3702(36.4)	1477(44.2)	1777(40.9)	448(14.9)	505.25	<0.0001
	Yes	6134(63.6)	3717(66.2)	2234(30.3)	183(3.6)		
Employment status	No	6760(69.6)	3207(52.5)	2965(37.1)	588(10.4)	331.75	<0.0001
	Yes	3076(30.4)	1987(71.2)	1046(27.2)	43(1.6)		
Annual household income	1st quintile	2158(20.2)	814(43.3)	1135(44.7)	209(12.0)	369.64	<0.0001
	2nd quintile	2087(20.0)	925(48.3)	1031(43.7)	131(8.0)		
	3rd quintile	2032(20.1)	1159(61.8)	757(31.5)	116(6.7)		
	4th quintile	1846(20.0)	1187(69.1)	579(25.9)	80(5.0)		
	5th quintile	1713(19.8)	1109(68.6)	509(24.6)	95(6.8)		
Household structure	Living alone	2491(24.0)	1038(46.5)	1208(41.7)	245(11.8)	230.16	<0.0001
	Living with a spouse	4832(48.6)	2833(64.0)	1840(31.9)	159(4.1)		
	Living with children	2513(27.4)	1323(58.0)	963(31.5)	227(10.5)		

^1^ Percentage adjusted with individual sampling weight.

**Table 2:** Mean and standard deviation of MMSE and significance test of mean difference in MMSE among categories of each variable by age group ^1^.

Variables	Categories	Age Group								
		65–74			75–84			85+		
		M ± SD ^1^	t or F	*p*-Value	M ± SD	t or F	*p*-Value	M ± SD	t or F	*p*-Value
Total		26.25 ± 0.05	-	-	24.27 ± 0.07	-	-	21.79 ± 0.21	-	-
Demographic-sociological characteristics										
Gender	Male	26.81 ± 0.06	10.75	<0.0001	25.59 ± 0.09	17.61	<0.0001	24.01 ± 0.29	8.74	<0.0001
	Female	25.83 ± 0.06			23.30 ± 0.09			20.53 ± 0.27		
Education	Uneducated	23.40 ± 0.14	313.98	<0.0001	21.85 ± 0.11	315.02	<0.0001	19.37 ± 0.27	64.44	<0.0001
	Elementary	25.85 ± 0.07			24.55 ± 0.10			23.07 ± 0.38		
	Middle-high	27.23 ± 0.06			26.19 ± 0.11			24.92 ± 0.40		
	College or above	28.20 ± 0.11			27.15 ± 0.20			25.61 ± 0.61		
Spouse	No	25.58 ± 0.10	−8.34	<0.0001	23.22 ± 0.11	−13.54	<0.0001	20.94 ± 0.27	−7.02	<0.0001
	Yes	26.51 ± 0.05			25.08 ± 0.08			23.82 ± 0.31		
Employment status	No	26.16 ± 0.06	−2.74	0.0061	24.07 ± 0.08	−5.53	<0.0001	21.72 ± 0.22	−1.75	0.0806
	Yes	26.41 ± 0.07			24.88 ± 0.12			22.94 ± 0.67		
Annual household income	1st quintile	25.22 ± 0.14	41.35	<0.0001	23.38 ± 0.13	17.76	<0.0001	21.40 ± 0.34	2.27	0.0591
	2nd quintile	25.99 ± 0.11			24.49 ± 0.13			22.47 ± 0.39		
	3rd quintile	26.02 ± 0.10			24.80 ± 0.15			22.45 ± 0.52		
	4th quintile	26.49 ± 0.09			24.26 ± 0.18			20.55 ± 0.70		
	5th quintile	27.08 ± 0.08			24.84 ± 0.19			21.96 ± 0.58		
Household structure	Living alone	25.65 ± 0.12	18.59	<0.0001	23.60 ± 0.13	73.19	<0.0001	21.98 ± 0.32	27.02	<0.0001
	Living with a spouse	26.43 ± 0.06			25.15 ± 0.09			23.87 ± 0.32		
	Living with children	26.33 ± 0.09			23.47 ± 0.15			20.16 ± 0.39		
Health status										
Subjective health status	Bad	25.36 ± 0.09	96.2	<0.0001	23.48 ± 0.10	71.32	<0.0001	20.96 ± 0.31	8.48	0.0002
	Moderate	26.38 ± 0.09			24.65 ± 0.13			22.17 ± 0.45		
	Good	26.88 ± 0.06			25.27 ± 0.11			22.90 ± 0.36		
Number of chronic diseases	0	26.96 ± 0.12	50.41	<0.0001	25.21 ± 0.26	15.6	<0.0001	21.55 ± 0.92	0.29	0.7477
	1	26.84 ± 0.09			24.90 ± 0.18			22.18 ± 0.55		
	2 or more	25.95 ± 0.06			24.09 ± 0.08			21.75 ± 0.24		
Depression	No	26.48 ± 0.05	9.67	<0.0001	24.71 ± 0.08	10.61	<0.0001	22.18 ± 0.26	2.63	0.0086
	Yes	25.09 ± 0.14			23.03 ± 0.14			21.00 ± 0.36		
Health behavior										
Nutrition Management	Low risk	26.88 ± 0.06	99.32	<0.0001	25.38 ± 0.11	80.93	<0.0001	23.86 ± 0.43	18.37	<0.0001
	Medium risk	25.94 ± 0.07			24.12 ± 0.10			21.86 ± 0.31		
	High risk	24.98 ± 0.15			23.13 ± 0.15			20.46 ± 0.36		
Smoking	No	26.22 ± 0.05	−2.3	0.0217	24.22 ± 0.07	−2.91	0.0036	21.76 ± 0.22	−0.85	0.3944
	Yes	26.53 ± 0.13			24.88 ± 0.22			22.48 ± 0.81		
Drinking	No	26.09 ± 0.06	15.74	<0.0001	23.98 ± 0.08	39.77	<0.0001	21.62 ± 0.23	4.92	0.0073
	Ordinary	26.65 ± 0.10			25.17 ± 0.19			22.00 ± 0.68		
	Excessive	26.59 ± 0.11			25.52 ± 0.19			24.08 ± 0.75		
Exercise	No	25.69 ± 0.09	36.79	<0.0001	23.32 ± 0.12	69.95	<0.0001	20.65 ± 0.31	15.52	<0.0001
	Insufficient	26.06 ± 0.11			24.05 ± 0.15			22.41 ± 0.39		
	Satisfactory	26.61 ± 0.06			25.10 ± 0.09			23.31 ± 0.39		
Dementia screening	No	26.23 ± 0.06	−0.83	0.4071	23.85 ± 0.10	−6.33	<0.0001	21.28 ± 0.29	−2.84	0.0046
	Yes	26.31 ± 0.08			24.71 ± 0.09			22.48 ± 0.32		
Function status										
IADL	Full independence	26.57 ± 0.04	−14.4	<0.0001	25.30 ± 0.07	−21.95	<0.0001	23.76 ± 0.30	−7.74	<0.0001
	Need help	23.98 ± 0.17			22.11 ± 0.13			20.63 ± 0.27		
APME	No	24.50 ± 0.17	−11.32	<0.0001	22.64 ± 0.14	−14.64	<0.0001	20.55 ± 0.30	−6.32	<0.0001
	Yes	26.47 ± 0.05			24.92 ± 0.07			23.14 ± 0.28		
Social relations										
Relatives contact frequency	Hardly ever	25.71 ± 0.17	10.02	<0.0001	23.35 ± 0.19	30.27	<0.0001	20.94 ± 0.40	3.93	0.0196
	<1 a week	26.23 ± 0.05			24.28 ± 0.08			22.16 ± 0.26		
	≥1 a week	26.57 ± 0.10			25.30 ± 0.17			22.86 ± 0.88		
Acquaintances contact frequency	Hardly ever	24.59 ± 0.19	48.13	<0.0001	22.94 ± 0.18	39.66	<0.000	20.06 ± 0.38	18.98	<0.0001
	<1 a week	26.25 ± 0.10			24.20 ± 0.15			22.53 ± 0.39		
	≥1 a week	26.50 ± 0.05			24.69 ± 0.08			22.94 ± 0.30		
Activities										
Watching TV	<5 h a day	26.40 ± 0.05	4.84	<0.0001	24.43 ± 0.08	3.24	0.0012	21.80 ± 0.26	0.05	0.9639
	≥5 h a day	25.89 ± 0.09			23.97 ± 0.12			21.78 ± 0.37		
Travel	No	25.87 ± 0.06	−11.13	<0.0001	23.94 ± 0.08	−7.9	<0.0001	21.30 ± 0.23	−5.71	<0.0001
	Yes	26.87 ± 0.06			25.10 ± 0.12			24.23 ± 0.46		
Leisure	No	25.85 ± 0.14	−3.15	0.0016	23.44 ± 0.19	−4.81	<0.0001	20.14 ± 0.53	−3.61	0.0003
	Yes	26.32 ± 0.05			24.23 ± 0.07			22.24 ± 0.22		
Learning	No	26.17 ± 0.05	−5.62	<0.0001	24.14 ± 0.07	−5.52	<0.0001	21.65 ± 0.23	−2.73	0.0064
	Yes	26.84 ± 0.11			25.09 ± 0.16			23.33 ± 0.57		
Club	No	26.15 ± 0.05	−12	<0.0001	24.20 ± 0.07	−7.95	<0.0001	21.73 ± 0.22	−3.57	0.0004
	Yes	27.90 ± 0.14			26.89 ± 0.33			26.28 ± 1.25		
Frequency of social activities	No	25.48 ± 0.08	106.46	<0.0001	23.50 ± 0.08	194.82	<0.0001	21.13 ± 0.23	61.63	<0.0001
	<1 a week	26.73 ± 0.05			25.76 ± 0.11			25.68 ± 0.38		
	≥1 a week	27.37 ± 0.14			26.90 ± 0.23			26.05 ± 0.89		
Religion	No	26.06 ± 0.08	−3.22	0.0013	23.92 ± 0.11	−4.13	<0.0001	20.76 ± 0.36	−3.86	0.0001
	Yes	26.37 ± 0.06			24.50 ± 0.09			22.47 ± 0.26		
SCC	No	26.41 ± 0.05	8.23	<0.0001	24.56 ± 0.08	6.72	<0.0001	22.04 ± 0.28	1.75	0.0802
	Yes	25.37 ± 0.12			23.61 ± 0.11			21.31 ± 0.31		
Electronic device -based activities	Zero	24.39 ± 0.11	306.21	<0.0001	22.78 ± 0.09	447.12	<0.0001	20.95 ± 0.24	63.49	<0.0001
	One	25.74 ± 0.10			25.25 ± 0.12			23.91 ± 0.49		
	Two more	27.19 ± 0.05			26.87 ± 0.10			26.66 ± 0.48		

MMSE = mini mental status examination; IADL = instrumental activity of daily life; APME = ability to perform muscular exercise; SCC = use of senior citizen center; M = mean; SD = standard deviation. ^1^ Mean and standard deviation adjusted with individual sampling weight.

**Table 3:** Final regression models on MMSE after controlling the sociodemographic characteristics by age group ^1^.

Variables	Categories	Age Group								
		65–74			75–84			85+		
		*β* ^1^	t ^1^	*p*-Value	*β*	t	*p*-Value	*β*	T	*p*-Value
Intercept		21.69	72.17	<0.0001	20.05	76.06	<0.0001	18.6	26.97	<0.0001
Gender	Male									
	Female	−0.23	−2.67	0.0076	−0.43	−2.97	0.0029	−0.94	−1.86	0.0639
Education	Uneducated									
	Elementary	1.6	10.34	<0.0001	1.62	10.65	<0.0001	2.89	6.05	<0.0001
	Middle-high	2.48	15.43	<0.0001	2.5	14.3	<0.0001	4.11	7.36	<0.0001
	College or above	2.95	14.98	<0.0001	2.77	10.21	<0.0001	4.29	5.52	<0.0001
Spouse	No									
	Yes	0.21	1.17	0.2436	0.54	2.21	0.0275	1.28	1.41	0.1586
Employment status	No									
	Yes	0.09	1.05	0.2947	0.32	2.69	0.0073	0.22	0.35	0.725
Annual household income	1st quintile									
	2nd quintile	0.33	2.21	0.0272	0.22	1.34	0.179	0.55	1.1	0.2734
	3rd quintile	0	−0.01	0.9922	0.03	0.15	0.8846	0.67	1.23	0.218
	4th quintile	0.18	1.09	0.2744	−0.02	−0.07	0.9405	−0.77	−1.18	0.2404
	5th quintile	0.35	2.1	0.0358	0.2	0.82	0.4098	0.7	0.89	0.3753
Household structure	Living alone									
	Living with a spouse	−0.38	−1.8	0.0723	−0.49	−1.8	0.0725	−1.76	−1.85	0.0654
	Living with children	−0.31	−1.6	0.1087	−0.77	−3.39	0.0007	−1.64	−2.82	0.005
Nutrition management	Low risk									
	Medium risk	−0.26	−2.99	0.0028						
	High risk	−0.61	−4.4	<0.0001						
Dementia screening	No									
	Yes	0.32	3.72	0.0002	0.68	6.1	<0.0001			
IADL	Need help									
	Full independence	0.88	5.37	<0.0001	1.32	9.15	<0.0001			
APME	No									
	Yes	0.63	3.79	0.0002	0.7	4.91	<0.0001	1.17	3.06	0.0023
Relative contact frequency	Hardly ever									
	<1 a week				0.23	1.36	0.175			
	≥1 a week				0.84	3.83	0.0001			
Acquaintances contact frequency	Hardly ever									
	<1 a week	0.7	3.73	0.0002				1.36	2.99	0.0029
	≥1 a week	0.74	4.2	<0.0001				1.55	3.56	0.0004
Travel	No									
	Yes	0.25	2.96	0.0031				1.43	3.21	0.0014
Club	No									
	Yes	0.41	2.96	0.003						
Frequency of social activities	No									
	<1 a week				0.46	3.64	0.0003			
	≥1 a week				0.83	3.53	0.0004			
Religion	No									
	Yes				0.33	2.78	0.0055	1.11	3.1	0.002
SCC	No									
	Yes	−0.38	−3.28	0.001						
Electronic device-based activities	Zero									
	One	0.69	5.15	<0.0001	1.25	8.69	<0.0001			
	Two or more	1.16	9.72	<0.0001	1.67	10.44	<0.0001			
F(*p*-value)		58.47(<0.0001)			83.78(<0.0001)			25.08(<0.0001)		
$R^{2}$		0.28			0.35			0.38		
Adjusted $R^{2}$		0.28			0.35			0.37		

MMSE = mini mental status examination; IADL = instrumental activity of daily life; APME = ability to perform muscular exercise; SCC = use of senior citizen center; M = mean; SD = standard deviation. ^1^ Regression coefficients and t-statistics adjusted with individual sampling weight.

## Data Availability

Data are available with permission from the Health and Welfare Data Portal https://data.kihasa.re.kr/ (1 August 2021).
